# New insights regarding HCV-NS5A structure/function and indication of genotypic differences

**DOI:** 10.1186/1743-422X-9-14

**Published:** 2012-01-12

**Authors:** Lilian HT Yamasaki, Helen A Arcuri, Ana Carolina G Jardim, Cintia Bittar, Isabel Maria VG de Carvalho-Mello, Paula Rahal

**Affiliations:** 1 Department of Biology, Sao Paulo State University--UNESP, Sao Jose do Rio Preto, SP, Brazil; 2 Department of Clinical Medicine, Sao Paulo University--USP, Medicine College, Sao Paulo, SP, Brazil; 3 Viral Immunology Laboratory, Butantan Institute, Sao Paulo, SP, Brazil; 4 Rua Cristovao Colombo, 2265 Laboratorio de Estudos Genomicos, Jardim Nazareth, CEP: 15054-000, Sao Jose do Rio Preto, SP, Brazil

**Keywords:** Hepatitis C virus, Non-structural 5A protein, Bioinformatics, Genotype, Quasispecies, IFN response

## Abstract

**Background:**

HCV is prevalent throughout the world. It is a major cause of chronic liver disease. There is no effective vaccine and the most common therapy, based on Peginterferon, has a success rate of ~50%. The mechanisms underlying viral resistance have not been elucidated but it has been suggested that both host and virus contribute to therapy outcome. Non-structural 5A (NS5A) protein, a critical virus component, is involved in cellular and viral processes.

**Methods:**

The present study analyzed structural and functional features of 345 sequences of HCV-NS5A genotypes 1 or 3, using *in silico *tools.

**Results:**

There was residue type composition and secondary structure differences between the genotypes. In addition, second structural variance were statistical different for each response group in genotype 3. A motif search indicated conserved glycosylation, phosphorylation and myristoylation sites that could be important in structural stabilization and function. Furthermore, a highly conserved integrin ligation site was identified, and could be linked to nuclear forms of NS5A. ProtFun indicated NS5A to have diverse enzymatic and nonenzymatic activities, participating in a great range of cell functions, with statistical difference between genotypes.

**Conclusion:**

This study presents new insights into the HCV-NS5A. It is the first study that using bioinformatics tools, suggests differences between genotypes and response to therapy that can be related to NS5A protein features. Therefore, it emphasizes the importance of using bioinformatics tools in viral studies. Data acquired herein will aid in clarifying the structure/function of this protein and in the development of antiviral agents.

## Introduction

Hepatitis C is a major health problem; it is highly prevalent worldwide and has a high probability of persistence [1,2]. Chronic persistence can result in cirrhosis and hepatocellular carcinoma [3,4]. Hepatitis C virus (HCV) is member of the Flaviviridae family within the *Hepacivirus *genus, although many of its features are distinct from other family members including the structural organization of the protein and the 5'-cap independent translation [5].

On the basis of viral variability, HCV is classified into seven genotypes and more than 50 subtypes [6]. In addition, an infected patient will harbour different but related viral genomes known as quasispecies. This high variability can be explained by a combination of three factors: (1) viral RNA polymerase acts without proof-reading [7]; (2) HCV has co-evolved with human populations for millions of years [8]; (3) the viral life cycle is fast, resulting in the production of approximately 1.3 × 1012 virions per patient per day [9].

The HCV RNA genome translates a polyprotein that is cleaved by viral and host proteases to generate ten structural and non-structural proteins [10-12]. Among the non-structural proteins, NS5A is a phosphoprotein critical for the HCV life cycle. It is composed of approximately 447 amino acids and may participate in viral RNA replication, modulation of cell signaling pathways, interferon response, pathogenesis and apoptosis regulation. Its enzymatic functions and its complete structure have yet to be elucidated. However, evidence suggests that it functions through interaction with other HCV and host cell proteins [13-16].

NS5A is divided into three domains [17,18]. Domain I contains a membrane binding domain [19,20] and a zinc finger domain that are essential for HCV replication [17]. Domain II and III are naturally unstructured, performing function by interacting with several proteins [15,21].

The importance of NS5A protein in disease caused by hepatitis C is unquestioned. However, difficulties with experimental methods used to determine the structures of highly flexible proteins have resulted in a poor understanding of the overall structure and functions of NS5A. Such difficulties have led to the development of bioinformatic tools that are helpful in obtaining reliable data for these types of proteins. *Ab initio *tools are also important for studying proteins with low or no homology, and can be used to compare them with experimentally determined structures.

In the present study, complete sequences from HCV NS5A genotypes 1 and 3 were analyzed. These sequences were obtained from Brazilian patients who showed different responses to Peginterferon (PegIFN) therapy. Using these sequences, the aims were to analyze structural and functional features. The knowledge obtained should aid in the design of new drugs and vaccines, and in developing other resources to improve HCV therapy.

## Results

### Amino acid composition and secondary structure of NS5A

**Figure 1:**
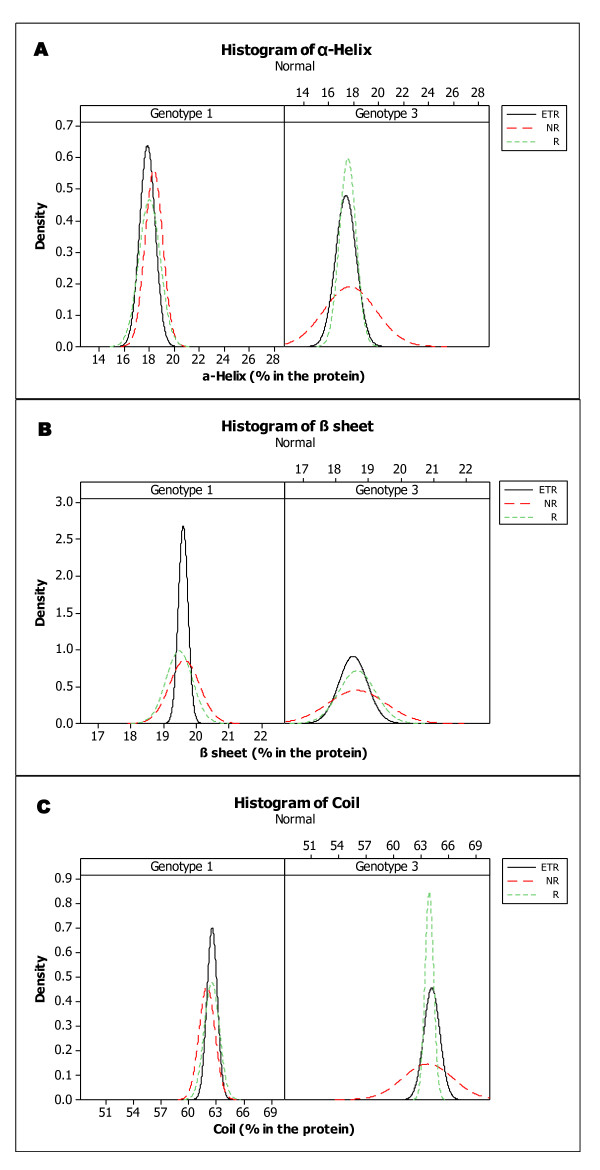
**Statistical analysis in second Structure composition (%) distribution of the NS5A sequences**. Plot: Difference in helix (**A**), sheet (**B**) and coil (**C**) percentage in the NS5A protein between genotype 1 and 3/therapy outcome. X-axis describes the percentage of each second structure type in total protein; y-axis means density (number of sequences with correspond percentage/total number of sequences) ETR-End of therapy responder, NR-nonresponder, R-responder.

**Figure 2:**
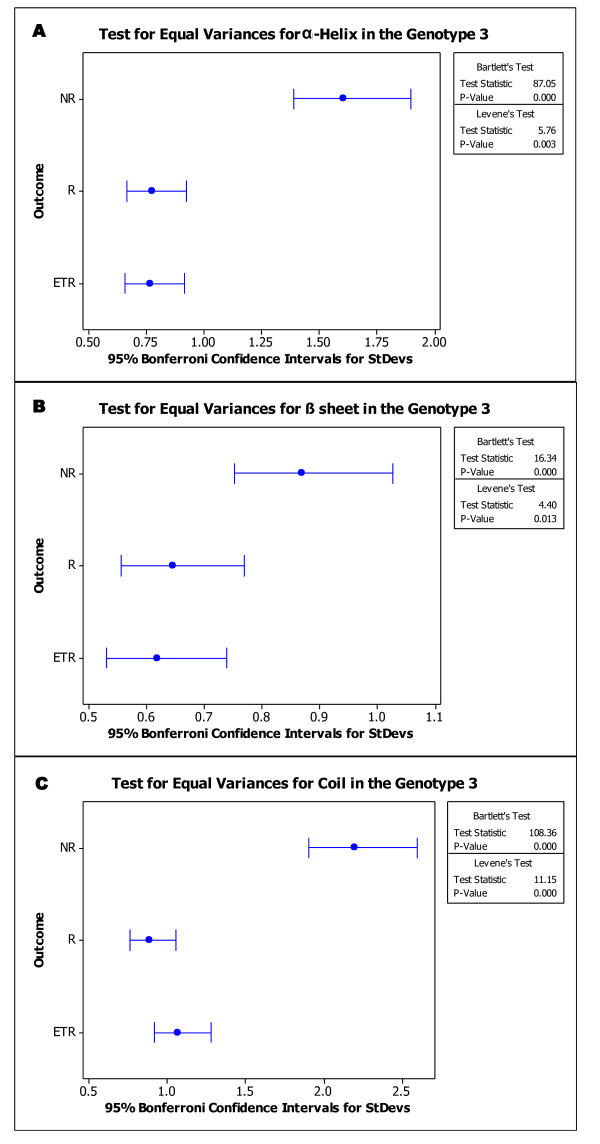
**Statistical analysis in second structure composition (%) variance of the NS5A genotype 3 sequences**. Plot: Statistical results for helix (**A**), sheet (**B**) and coil (**C**) variance in genotype 3. Square in the left shows the calculated values for each test. Note the variance different distribution in each response group. StDev-standard deviation.

Therapy response did not differ according to the amino acids composition or the secondary structure type composition. However, considering the genotypes, the average percentages of alanine, glutamic acid, glutamine and tyrosine present in the NS5A protein were different between the genotypes 1a, 1b and 3. The average percentages of cysteine, valine and threonine differed between genotype 1 and 3. All sequences obtained from genotype 1b presented with 2% tyrosine (data not shown). Secondary structure analysis demonstrated that distribution of each secondary structure type followed a normal distribution. Statistical analysis (*t*-test) suggests that the three secondary arrangements are high significant factors (*p*-value < .001) to differ the genotypes. Composition of helix, sheet or coil in percentage did not result in significant difference when we compared these three arrangements the therapy outcome responses (Figure 1A-C). In contrast, when we compared the variation of these compositions among the response groups, there is a great (and statistical) difference, especially in genotype 3. Test for equal variances (Bartlett's test and Levene's test) resulted in different variation behaviour comparing the three outcome responses sequences in genotype 3 (Figure 2A-C). The same test applied for analysis intra-response group indicates that even when sequences are from different patients, if they are from the same therapy outcome group, they are not significantly different (*p *> .05) (Table 1).

**Table 1:** Statistical results (*t*-test) for comparison between genotype 1 and 3 secondary structure composition

*Genotype*	*No. of sequences (n)*	*Mean*	*StDev*	*SE Mean*	*p-value*
		***α-helix***			
1	109	18.06	0.52	0.05	0.00
3	143	17.51	0.41	0.03	

		***β-sheet***			
1	109	19.58	0.27	0.03	0.00
3	143	18.61	0.33	0.03	

		***Coil***			
1	109	62.35	0.52	0.05	0.00
3	143	63.88	0.38	0.03	

*StDev*: Standard Deviation, *SE*: Standard Error

### Transmembrane regions and pattern/motif search

**Figure 3:**
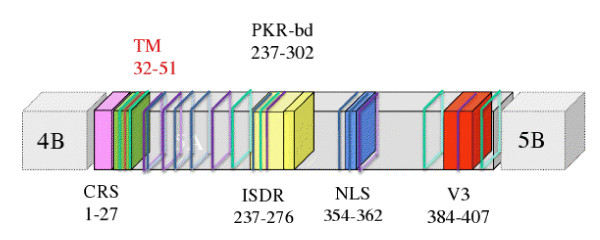
**Results of MEMSAT3 and Prosite**. Summary for NS5A reference AF009606. TM: transmembrane region, CRS: cytoplasmic retention site, PKR-bd: PKR binding site, NLS: nuclear localization site, V3: variable region 3. Lines-purple: n-glycosylation sites, blue: phosphorylation sites, green: n-myristoylation sites, orange: RGD site.

MEMSAT3 analysis demonstrated that all NS5A and reference sequences contained a possible transmembrane region between residues 32 and 51, and Prosite recognized seven functional patterns in each NS5A sequence. No relationship was observed between therapy response or genotype and pattern number or motif location. Table 2 presents a summary of each conserved pattern/motif encountered in the NS5A analysis. Between two and seven N-glycosylation sites were present within the same sequence. In two positions (69 and 268), this pattern was conserved in 90% of sequences; this motif was absent at these positions in 4% of cases. cAMP- and cGMP-dependent protein kinase phosphorylation sites were identified; 90% of sequences had a minimum of one such motif at position 357, but 4% of sequences from one patient lacked this motif. Protein kinase C (PKC) phosphorylation sites were present in all sequences at positions 71 and 238. The number of these sites in the same sequence ranged from five to eight. Casein kinase II phosphorylation sites were recognized in non-conserved positions, being present between 4 and 8 times in the same sequence. Tyrosine kinase phosphorylation sites were identified in a conserved position (residue 122 or 123). N-myristoylation sites were present in various numbers and several positions, with NS5A sequences possessing between four and eight of these motifs in the same sequence. A cell attachment sequence (RGD sequence) was present in all sequences, conserved in number and position; NS5A sequences possessed this motif at position 48. Figure 3 summarizes the results of the MEMSAT3 and Prosite analyses in the reference sequence AF009606.

**Table 2:** Conservation of patterns/motifs predicted on NS5A, described in percentage of sequences

*Motif/pattern name*	*Presence*	*Conserved Positions*	*No. of sites in same sequence*
**N-glycosylation**	95,65%	69, 268 (90%)	Two up to six

**cAMP/cGMP-dependent phosphorylation**	95,65%	357 (90, 14%)	One up to two

**PKC phosphorylation**	100%	71, 238 (98, 84%)	Five up to eight

**CKII phosphorylation**	100%	-	Four up to eight

**Tyrosine kinase phosphorylation**	99,99%	122 or 123(99, 99%)	Four up to eight

**N-myristoylation**	100%	-	Four up to eight

**Cell attachment sequence**	100%	48 (100%)	1

### Prediction of functional features

ProtFun analysis results are detailed in Table 3. It indicated that the complete NS5A has properties similar to proteins which play role in regulatory functions, replication and transcription, central intermediary metabolism or purines/pyrimidines. All sequences were classified as an enzyme of unknown class, with the transcription regulation as the gene ontology category. Calculated chi-square test showed significant difference (*p *< 0.05) between therapy outcome groups and between genotypes related to functional category predicted, with statistical power level varying from 0.617 to 1.

**Table 3:** Statistical results for comparison between therapy outcome and genotype in ProtFun analysis

*Region*	*Feature*	*Comparison*	*Chi-square*	*p-value*	*Stat power*
**NS5A**	Functional category	Outcome	18.832	0.004	0.711
		R and NR	10.697	0.013	0.617
		R and ETR	15.279	0.002	0.627
		NR and ETR	5.103	0.164	0.676
		Genotype	218.730	0.000	1

**Domain I**	GO*	Outcome	1.370	0.504	0.694
		Genotype	51.316	0.000	0.999

**Domain II**	Functional category	Outcome	27.769	0.000	0.861
		R and NR	15.903	0.000	0.881
		R and ETR	16.776	0.000	0.581
		NR and ETR	0.241	0.623	0.663
		Genotype	21.755	0.000	0.780
	
	Non/enzyme	Genotype	22.711	0.000	0.801
	
	GO	Outcome	1.792	0.408	0.665
		Genotype	116.636	0.000	1

**Domain III**	GO*	Outcome	24.680	0.000	0.572
		R and NR	5.488	0.064	0.587
		R and ETR	11.307	0.010	0.616
		ETR and NR	12.374	0.006	0.609
		Genotype	54.217	0.000	0.998

*values < 05 were excluded for the statistical calculation

The results from NS5A domain I suggested that this region is related to the functional category of energy metabolism, with enzymatic activity from unknown class. For domain I, prediction of gene ontology category varied among transcription regulation, growth factor, immune response, transcription and none of these categories. To calculate the chi-square, categories below 5 sequences were excluded. Significant difference was observed between genotypes but not between therapy response groups, with statistical power of 0.999.

Prediction for domain II sequences varied in functional category, enzymatic function and gene ontology category. Resulted functional category includes energy metabolism and translation, with statistical difference between therapy outcome groups (except between non-responders--NR and end of therapy responders- ETR groups) and genotype. Enzymatic function prediction and gene ontology (GO) category prediction had significant difference between genotypes, with statistical power of 0.801 and 1, respectively.

For domain III analysis, all the sequences were predicted to be similar to proteins related to transport and binding, with nonenzyme function. The gene ontology category varied among growth factor, immune response, stress response, hormone, voltage-gated ion channel and unknown. These differences were significant different between end of therapy responders (ETR) sequences and the other two outcome groups (non-responders--NR, responders--R) and between genotypes. Statistical power values were 0.6 and 1, respectively.

## Discussion

Infection with genotype 1 results in lower therapy success rates than other genotypes [22]. No previous study was found connecting NS5A amino acids composition or secondary structure type (Figure 1, 2 and Table 1) to this difference in response rate. The present study suggests that these two characteristics present genotypic differences. These differences could affect NS5A functions, by modifying its interactions with other viral and host proteins, or its stability. Consequently, these differences could affect viral resistance, replication and other properties linked to NS5A that differ among genotypes.

Indeed, some observations of the viral genotype specific features are reported between genotype 1 and 2. Viral dynamics was the first property detected, in a study that collected viral load data in patients receiving IFN therapy. Viral kinetics was greatly different. In genotype 1 infected patients, IFN effectiveness, free virion clearance rate and cell death rate were lower than genotype 2 HCV hosts. In contrast, percentage of individuals that reached an undetectable level during 14 days of therapy was higher in genotype 2 infected individuals [23]. Our group also found *in vivo *indication of divergences in NS5A quasispecies composition and mutational profiles between genotypes 1 and 3 in baseline specimens [24,25]. Using the sequences derived from these studies, we showed that there is indication of structural and functional differences between NS5A-1a/b and NS5A-3 (Table 3). In addition, at least for genotype 3, there were differences in variance of structure between the different responses groups (Figure 2). This divergence is not observed when we compare the sequences extracted from patients with the same therapy response. Possibly resulting from the structural differences, the functional prediction profile was also different between genotypes.

*In vivo *and derived *in silico *results point to a relationship between NS5A and genotypic IFN response rate. However, *in vitro *researches results are still controversial. In 2008, it was reported that cells infected by 2a-NS5A-containing replicons presented lower degree of IFN antagonism than the counterpart containing 1b-NS5A. The same study also found suggestions that the V3 domain and the C-terminal region of the NS5A are related to IFN differential reaction [26]. Other study in 2010 did not reached similar conclusions. In this case, cells expressing 1b and 2a NS5A protein presented analogous capability of IFN responses inhibition and IL-8 expression induction. The author suggests that other viral factors and/or host factors may be involved in the genotypic difference of HCV [27]. Posterior, it was also described that HCV with recombinant NS5A from different genotypes presents different sensibility to NS5A inhibitors, but not for IFN [28]. These hypothesis does support our group results, since it seems that NS5A has still numerous unknown direct and indirect interactions with host and viral proteins, consequence from the high structural flexibility observed in domains II and III of the protein [15,21].

High structural flexibility is the key of the multifunctionality in promiscuous proteins [29]. The NS5A protein, as other promiscuous proteins, presents an intrinsic disorder. Recent studies showed that the domain III can be unfold or partially fold in helix [21], another feature of promiscuous proteins, which can present different conformations depending on the interactions made [29]. Our secondary structure prediction shows a high percentage of coils in NS5A, which results in the high structural flexibility of the NS5A. In addition with ProtFun predictions, we observed that NS5A may have genotypic differences in performed functions, varying also between the domains, with a great statistical difference (*p *< .001) and power (> .95) (Table 3). Despite of the essential functions for HCV infection, NS5A from different genotypes may have different secondary functions. These functions may lead directly or indirectly to the different SVR rate in genotypes. Also, it would explain why NS5A from different genotypes have different behaviour when the mutational and quasispecies profile are analysed [24,25].

Prosite analysis demonstrated the presence of several potential co-and post-translational modification sites that are well conserved in the sequence, including N-glycosylation sites (Figure 3 and Table 2). Carbohydrate binding can confer a different function on a protein. For example, it can lead to the addition of epitopes that facilitate the recognition of other proteins [30,31]. There are no studies that describe glycosylation of NS5A, but in some viruses, glycosylated proteins can be essential for viral assembly [32]. Helenius (1994) demonstrated that glycosylation promoted an increase in solubility, and possibly in interactions with chaperones on the endoplasmatic reticulum, thereby affecting folding and stabilization of the protein. Proteins without this modification could assemble in a non-reversable form or exit the endoplasmatic reticulum [33]. N- and O-linked glycosylation are also described in non-reticulum compartments, such as nucleus and cytosol [34]. Since this discover, several cytosolic proteins involved in the process of adding carbohydrate to proteins were characterized [35,36]. Proteins which undergo these unusual glycosylation processes are linked with several functions, including nuclear membrane structure and transcription factors [37].

Following this hypothesis, NS5A glycosylation may be essential for maintaining its functional structure, as these modifications sequence appear to be conserved. In addition, this modification may play an important role in nuclear localization of NS5A mutants.

Potential phosphorylation sites were identified in NS5A. This modification has been experimentally described and is important for interaction with core proteins and for viral assembly [38]. Phosphorylation is a reversible modification process, and may be key to the multifunctionality of NS5A. Several proteins were identified as playing a role in NS5A phosphorylation including AKT, p70s6K, MEK, MKK1, CKI, CKIIe and Syk [39-41] but we found no study has described phosphorylation by PKC or cAMP-/cGMP-dependent protein kinase. These proteins are still candidates for this process, as details concerning NS5A phosphorylation have yet to be fully elucidated.

Possible myristoylation sites with qualitative conservation were recognized in NS5A. Covalent myristate binding is not reversible and alters the protein's hydrophobicity. In viral proteins such as Arenavirus and Arterivirus, this modification is related to functions such as protein cell localization and protein-protein interactions [42,43]. There are no studies describing myristoylation in the NS5A protein. However, we suggest that this process is important in structural/functional stabilization of NS5A. If experimental data demonstrated that these modifications are present in NS5A, these sites could be possible targets for new antiviral agents.

Interestingly, a cell attachment site (RGD) was present in all sequences between residues 48 and 52. This region is inside the trasmembrane region predicted by MEMSAT3. RGD is a sequence for interaction with integrins, proteins located on the cell surface that act on cell-cell and cell-extracellular matrix interactions [44]. Although the intracellular functions of RGD require further investigation, studies concerning proprotein convertase 1 showed that this sequence is essential for correct folding in the endoplasmatic reticulum and transport to secretory glands [45].

Micelles with cyclic RGD peptides transfected into HeLa cells tend to congregate in the perinuclear region [46]. Therefore, the RGD sequence in NS5A genotypes 1 and 3 could have a role in (1) folding and intracellular transport and/or (2) nuclear and perinuclear localization. The NS5A protein has a functional nuclear location signal (NLS) at its carboxy terminal [47]. The complete protein form is predominantly located in the cytoplasm and/or in the perinuclear region [47,48]. However, forms in which the NS5A amino terminal region (residues 1-31) has been deleted predominate in the nucleus [49]. These deleted forms occur naturally during infection, resulting in cell caspase activity [50,51]. The function of these nuclear forms requires further study but they have been shown to be transcriptional regulators [52-54]. Furthermore, these forms can transport other proteins complexed with NS5A. The c-Raf protein interacts with the NS5A carboxy terminal and is detected in the cell nucleus with these deleted forms of the NS5A protein [54].

Regarding these studies, we suggest that the factors participating in NS5A nuclear localization are (1) deletion of the amino terminal region, which inhibits the NLS region; (2) presence of the NLS without mutations; (3) possible interactions between RGD and proteins related to transport through the nuclear envelope (4) possible glycosylation of NS5A similar to other nuclear functional proteins.

It is important to highlight that if other proteins are transported to the nucleus with c-Raf, nuclear NS5A could be important to the regulation and modulation of cell processes.

## Conclusions

This research presents new insights regarding HCV NS5A genotypes 1 and 3. In addition, it demonstrates the importance of applying bioinformatic tools to the study of proteins that are difficult to investigate by other experimental procedures. There was no relationship between the response to therapy and primary structure, but for genotype 3 secondary structure variances were different between the three outcome groups. In addition, there is evidence that the primary/secondary structure differs among genotypes and that this could be important during the infection process. Functional prediction also indicated that NS5A may have functional difference between genotypes. Altogether, structural and functional properties show that the two genotypes behaviour during infection have differences. The acquired data can be compared with future experimental data regarding the NS5A protein and may help in developing new antiviral strategies, considering the genotypic differences present in Hepatitis C virus.

## Materials and methods

### Sequence bank

The sequence bank included 345 NS5A complete sequences that were obtained from previous studies by our group [24,25] and nine reference sequences from Genbank. These sequences were extracted from 23 Brazilian patients infected with HCV genotypes 1a, 1b or 3. The accession numbers in Genbank are EU309511aEU309673 for genotype 1; EU826174 to EU826233 and from EU826249 to EU826352 for genotype 3. These samples comprised patients who had a sustained virological response (SVR), non response (NR) or end of treatment response (ETR) after conventional therapy based on Interferon (genotype 3) or Peginterferon (genotype 1) plus Ribavirin. Details of the study population are presented in Table 4. Redundant amino acid sequences were excluded, using the software LOCQSPEC 1.0 [55], resulting in 252 different sequences of complete NS5A.

**Table 4:** Characteristics of the study population and number of NS5A different sequences

*Patient #*	*Genotype*	*Therapy outcome*	*No. of sequences*
RF03	1A	ETR	12
RF37	1A	ETR	11
RF42	1B	ETR	14
RF45	1A	ETR	13
RF16	1A	NR	13
RF22	1B	NR	5
RF39	1B	NR	3
RF44	1B	NR	11
RF05	1B	R	12
RF35	1A	R	10
RF40	1A	R	5
RF20	3	ETR	14
RF31	3	ETR	10
RF109	3	ETR	13
RF119	3	ETR	12
RF07	3	NR	12
RF60	3	NR	8
RF75	3	NR	12
RF145	3	NR	10
RF80	3	R	14
RF15	3	R	10
RF18	3	R	15
RF59	3	R	13
TOTAL	-	-	252

### Amino acids and secondary structure analysis

The percentage of each amino acid type was calculated and secondary structures investigated in all complete NS5A sequences from the sequence bank. These calculations were performed using the PROF program [56], using the Predict Protein Server http://www.predictprotein.org[57].

### Transmembrane region prediction

Prediction of transmembrane regions was developed by the MEMSAT3 program [58]http://bioinf.cs.ucl.ac.uk/psipred/. All NS5A sequences from the sequence bank were analyzed using this program.

### Prediction of sites

All sequences were analyzed using the PROSITE program [59], from the Predict Protein Server [57]. Prosite is a pattern data bank, based on scientific publications or research describing the function of determined protein groups [59].

### Prediction of functional features

Sequences were submitted to ProtFun 2.2 Server. The method is based on sequence derived protein features such as predicted post translational modifications, protein sorting signals and physical/chemical properties calculated from amino acid composition. This allows prediction of functionality for proteins which no homology can be found [60,61]. Acquired data were organized in tables (not shown) to posterior statistical analysis.

### Statistical analysis

In order to establish if there were differences between the prediction results between the response groups or genotypes, test of homogeneity (chi-square test), *t*-test and equal variance test was calculated using the software MiniTab^® ^15 (Minitab Inc., USA). Values under 5 (five) were excluded from statistical calculation, since these results could be deviation from the sample. Statistical power calculation was performed using online software Russ Lenth's power and sample size page [62].

## Competing interests

The authors declare that they have no competing interests.

## Authors' contributions

LHTY carried out all experiments, acquisition of data, analysis and interpretation of data, and drafting the manuscript; HAA helped with the acquisition of data and interpretation of results; ACGJ, CB and IMVGCM participated in the study and the writing of the manuscript, PR conceived the study, participated in its analysis and coordination, and supplied suggestions for this manuscript. All authors read and approved the final manuscript.
